# Supplementary material for: The Effects of Mycovirus BmPV36 on the Cell Structure and Transcription of Bipolaris maydis
Source: J Fungi (Basel). 2024 Feb 6;10(2):133. doi: 10.3390/jof10020133 (PMC10890528; doi:10.3390/jof10020133)

Table S1. Information of primers.

Gene Name	Primer Name	Sequences（5 '- 3'）
Actin	Act-F	TCAAGATCATCGCTCCTCCC
	Act-R	GGACCGCTCTCGTCGTACTC
COCC4DRAFT_82543	Co43-F	GTTTCCTTATGGGTTTCGG
	Co43-R	ACACGCCAGCACCAGTC
COCC4DRAFT_80536	Co36-F	AAATCGGTGGACTGGTTG
	Co36-R	GATCCTTGATGGCCTTGT
COCC4DRAFT_123791	Co91-F	GACTTGAGCGACATGGAGG
	Co91-R	CCTGGCAGAATGTTGGACT
COCC4DRAFT_50090	Co90-F	TGCTCGTTTCACTAGGTGT
	Co90-R	GGTTGGTGGTCGTTTATG
COCC4DRAFT_29453	Co53-F	GATGCTGGCACTCCACTCT
	Co53-R	CAGGAAGCGAAATGGTAGG
COCC4DRAFT_53511	Co11-F	GACAACGGCTTCGTAGACC
	Co11-R	GGCAGCAGCGTAAGTGAG
COCC4DRAFT_175201	Co01-F	CGTAGGTGGAAACGGTAA
	Co01-R	TGGTCTCGCCATAGATTGT
COCC4DRAFT_18807	Co07-F	AGCAGTGCGAGCGAGTT
	Co07-R	TTTCCAAATGCCTTCTTCA

Table S2. Biological characteristics of BmPV36-carring *B. maydis* and virus free *B. maydis*.

	Diameter(cm)	Sporulation quantity 10^4 spores/mL	Disease index
BM36	5.35±0.06 a	7.53 ±0.16 b	25.35±0.5 b
BM36-Non	5.55±0.04 a	62.3±2.60 a	65.25±0.6 a

Note: Different lowercase letters within a column indicate a significant difference in a variable among sites ($P < 0.05$) according to Tukey's test. BM36: BmPV36-carring *B. maydis;* BM36-Non: virus free *B. maydis.*

Table S3. Differentially expressed genes of major facilitator superfamily (MFS).

Gene name	Gene description	Log2FC (BM2 / BM-Non2)	Log2FC (BM5 / BM-Non5)
COCC4DRAFT_82543	MFS domain-containing protein	0.361	0.145
COCC4DRAFT_80536	MFS domain-containing protein	0.312	0.174
COCC4DRAFT_81052	MFS domain-containing protein	0.372	0.193
COCC4DRAFT_206131	MFS general substrate transporter	0.164	0.210
COCC4DRAFT_50623	MFS domain-containing protein	0.157	0.242
COCC4DRAFT_58539	MFS domain-containing protein	0.438	0.249
COCC4DRAFT_65994	MFS domain-containing protein	0.065	0.263
COCC4DRAFT_175095	MFS domain-containing protein	0.309	0.2755
COCC4DRAFT_151949	MFS domain-containing protein	0.196	0.361
COCC4DRAFT_89192	MFS domain-containing protein	0.106	0.348
COCC4DRAFT_191448	MFS domain-containing protein	0.195	0.364
COCC4DRAFT_35339	MFS domain-containing protein	0.375	0.392
COCC4DRAFT_132658	MFS domain-containing protein	0.108	0.403
COCC4DRAFT_132539	MFS domain-containing protein	0.377	0.454
COCC4DRAFT_154131	MFS domain-containing protein	0.306	0.461
COCC4DRAFT_75577	MFS domain-containing protein	0.132	0.465
COCC4DRAFT_147715	MFS domain-containing protein	0.397	0.477
COCC4DRAFT_53483	MFS domain-containing protein	0.405	0.528

Note: BM2: BmPV36-carring *B. maydis* cultured for 2 d; BM5: BmPV36-carring *B. maydis* cultured for 5 d; BM-Non2: Virus free *B. maydis* cultured for 2 d; BM-Non5: Virus free *B. maydis* cultured for 5 d.

Table S4. Differentially expressed genes of enzyme.

Gene name	Gene description	Log2FC (BM2 / BM-Non2)	Log2FC (BM5 / BM-Non5)
COCC4DRAFT_123791	geranylgeranyl diphosphate synthase (EC 2.5.1.29)	0.265	0.301
COCC4DRAFT_50090	Mevalonate kinase (MK) (EC 2.7.1.36)	0.869	0.428
COCC4DRAFT_29453	Cellulase D (EC 3.2.1.4)	0.687	0.290
COCC4DRAFT_140403	Cellulase D (EC 3.2.1.4)	0.956	0.416
COCC4DRAFT_88117	Cellulase D (EC 3.2.1.4)	0.668	0.434
COCC4DRAFT_124946	Cellulase D (EC 3.2.1.4)	0.132	0.438
COCC4DRAFT_53511	Cellulase D (EC 3.2.1.4)	0.894	0.457
COCC4DRAFT_175201	Pectinesterase (EC 3.1.1.11)	0.18	0.336
COCC4DRAFT_18807	Cutinase (EC 3.1.1.74)	0.576	0.256

Note: BM2: BmPV36-carring *B. maydis* cultured for 2 d; BM5: BmPV36-carring *B. maydis* cultured for 5 d; BM-Non2: Virus free *B. maydis* cultured for 2 d; BM-Non5: Virus free *B. maydis* cultured for 5 d.

Figure S1. Principal coordinate analysis. The x- and y-axis represent the two selected principal coordinate axes; the percentage represents the explanatory value of the principal coordinate axes for the difference in sample composition; distances between symbols on the ordination plot reflect relative dissimilarities. BM2: BmPV36-carring *B. maydis* cultured for 2 d; BM5: BmPV36-carring *B. maydis* cultured for 5 d; BM-Non2: Virus free *B. maydis* cultured for 2 d; BM-Non5: Virus free *B. maydis* cultured for 5 d.

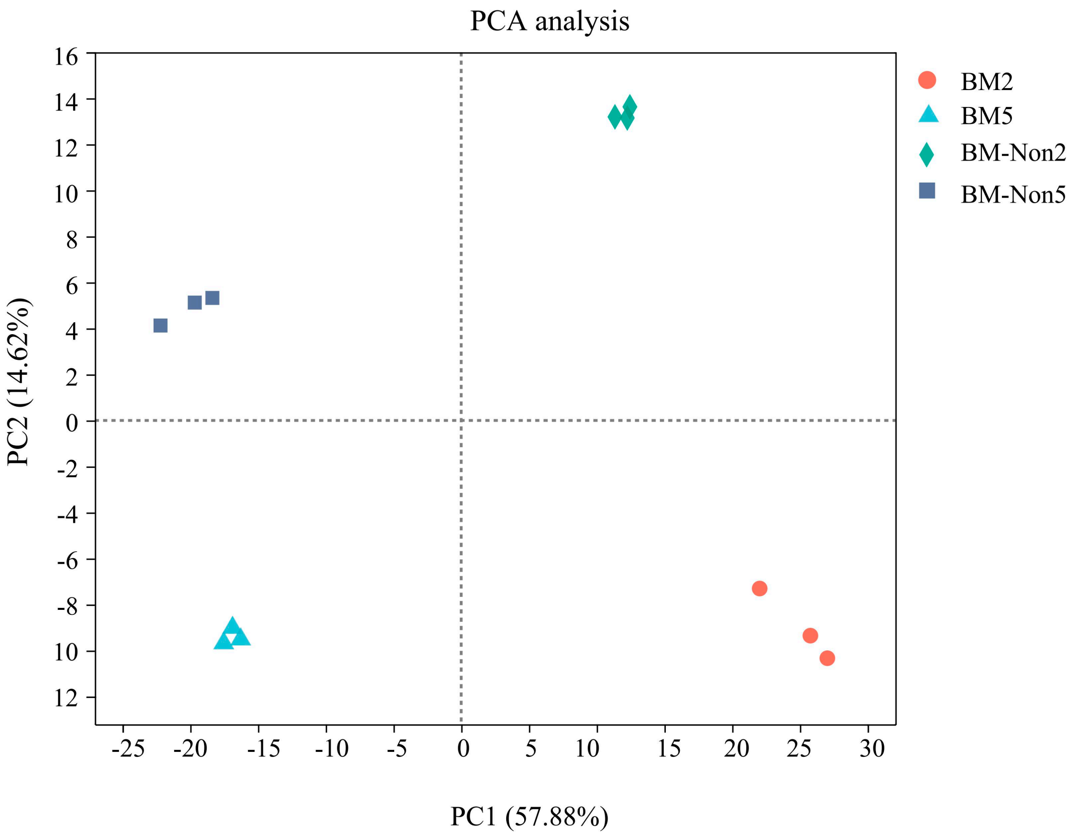

Figure S2. GO annotations analysis of all the differentially expressed genes at Level 2.

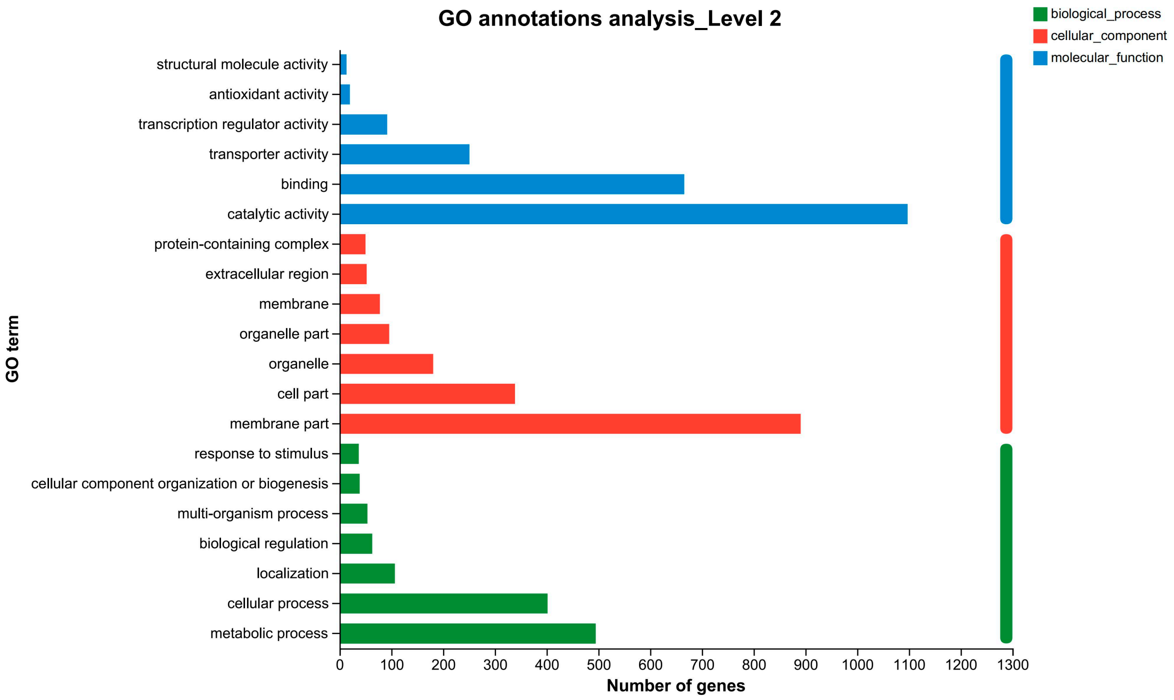

Figure S3. GO diagram of significantly over-represented GO terms in cell wall.

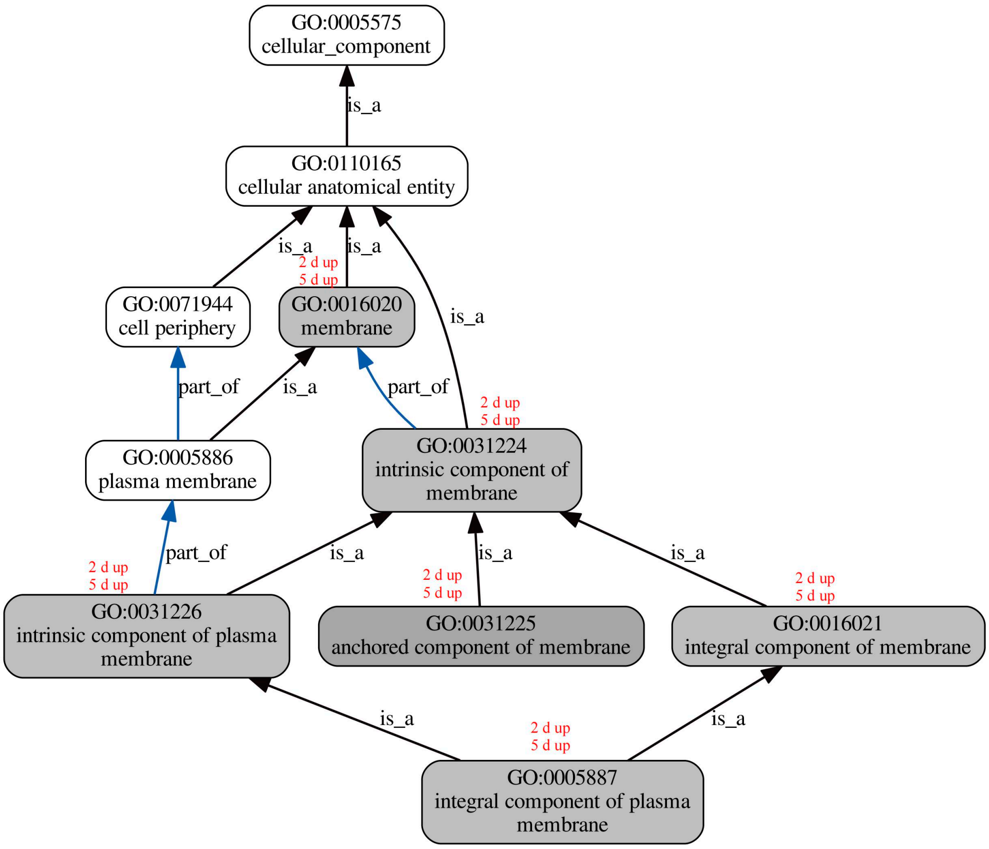

Supplement: Supplementary file 1 [file jof-10-00133-s001.zip › jof-2830744-supplementary.pdf]
